# Privacy-preserving GWAS analysis on federated genomic datasets

**DOI:** 10.1186/1472-6947-15-S5-S2

**Published:** 2015-12-21

**Authors:** Scott D Constable, Yuzhe Tang, Shuang Wang, Xiaoqian Jiang, Steve Chapin

**Affiliations:** 1 Department of EECS, Syracuse University, South Crouse Avenue, 13244 Syracuse, NY USA; 2 Department of Biomedical Informatics, University of California, San Diego, 9500 Gilman Drive, MC 0728, 92093 La Jolla, CA USA

**Keywords:** Genomic data privacy protection, GWAS, Statistical analysis, Secure multi-party computation

## Abstract

**Background:**

The biomedical community benefits from the increasing availability of genomic data to support meaningful scientific research, e.g., Genome-Wide Association Studies (GWAS). However, high quality GWAS usually requires a large amount of samples, which can grow beyond the capability of a single institution. Federated genomic data analysis holds the promise of enabling cross-institution collaboration for effective GWAS, but it raises concerns about patient privacy and medical information confidentiality (as data are being exchanged across institutional boundaries), which becomes an inhibiting factor for the practical use.

**Methods:**

We present a privacy-preserving GWAS framework on federated genomic datasets. Our method is to layer the GWAS computations on top of secure multi-party computation (MPC) systems. This approach allows two parties in a distributed system to mutually perform secure GWAS computations, but without exposing their private data outside.

**Results:**

We demonstrate our technique by implementing a framework for minor allele frequency counting and *χ*^2 ^statistics calculation, one of typical computations used in GWAS. For efficient prototyping, we use a state-of-the-art MPC framework, i.e., Portable Circuit Format (PCF) [1]. Our experimental results show promise in realizing both efficient and secure cross-institution GWAS computations.

## Introduction

As improving technology lowers the cost of genome sequencing, whole genome sequencing (WGS) data are becoming more affordable and accessible, enabling various new medical applications for precise diagnostics, personalized medicine, etc. Genome-wide association studies (GWAS), in particular, aim at better understanding the association between genetic variants and diseases by examining genetic mutations, which differ in a statistically significant way between individuals who have an illness and individuals who do not. Such analysis can benefit from using a large amount of sample data [2], which are usually present at multiple locations (especially in the case of rare disease studies) with independent administrative domains. For instance, these could be geographically distributed hospitals and medical institutions.

Due to the urgent needs of integrating and sharing genomic and medical data, various kinds of information networks emerged in different sectors and for various applications. For instance, eMERGE [3], pSCANNER [4] and PCORnet [5] have been established for accelerating scientific discovery and promoting personalized medicine. Health information exchange networks (e.g. NHIN [6], CommonWell [7] and GaHIN [8]) are being developed or used in practice for improving public health.

However, an inhibiting factor in cross-domain federated data analysis is increasing concern for individual privacy during biomedical information exchange. Without proper protection, individual-level information exchange can put patient-specific information at risk, which might have serious implications for research participants, such as discrimination for employment, insurance, or education [9]. In the U.S., privacy laws, such as HIPAA [10], have been established to protect individuals' medical data privacy. For example, under the HIPAA Safe Harbor rule,one must remove all the biometric identifiers to de-identify a dataset before sharing it. As genomic data are recognized by most researchers as a type of biometric identifiers, it is infeasible to directly de-identify or share raw genomic data under HIPAA regulation. In addition, NIH recently announced the Genome Data Sharing policy [11]to govern the sharing of genomic information based on the informed consent from patients and data-use agreements between data owner and users; sharing raw genomic data across institutions while complying with this privacy policy presents a challenge.

In this work, we focus on the privacy-preserving statistical analysis on federated genome datasets. Our aim is to enable genomic data possessing institutions to conduct collaborative GWAS without breaching any privacy agreements with their patients. Our primary approach is to apply recent advances in secure multiparty computation (MPC) [12,13] to the application domain of distributed GWAS analysis. In particular, recently developed MPC systems allow the expression of the secure computation in a high-level C-like programming language (with certain constraints). Based on the Portable Circuit Format (PCF) framework [1], a state-of-the-art MPC tool chain, we implemented two representative GWAS computations (i.e. minor allele frequency (MAF) counting and *χ*^2 ^statistics computation. Our system works for the two-party computation scenario, and its performance has been evaluated to be efficient for practical use in the presence of genome datasets with several hundreds of genotypes and standard Internet connections. When compared against noise-based protection [14-16], our approach does not sacrifice utility and can produce the computation accurate results under fixed-point approximation. Unlike distributed model learning frameworks proposed in [17-20], which exchange the aggregated intermediary statistics in plaintext, the proposed framework provides strong security protection through the extensive and intelligent use of cryptographic primitives (in the MPC runtime systems).

### Related work

Recently, practical MPC programming platforms have been developed for distributed computations involving two or three parties (e.g. PCF [1], Sharemind [21], Fair-play [12]) or more (e.g. GMW [22], FairplayMP [23]). Certain programming frameworks support high-level programming languages and compilers (e.g. Fair-play(MP) [12,23], SecreC [24], PCF [1]), while others expose a quite low-level circuit based interface (e.g. GMW [22]). Underneath, various techniques are used to ensure security, such as secret sharing [21], cryptography based garbled circuit, and oblivious transfer [1]. Other recent advances have focused on MPC techniques built upon the Fully Homomorphic Encryption (FHE) scheme proposed by Gentry [25]. However, pure FHE is known not to scale well enough to support practical MPC deployments; hence alternative protocols dubbed "Somewhat Homomorphic Encryption" (SHE) have been proposed [26-28]. Under certain assumptions, SHE can be much faster than FHE and without sacrificing privacy guarantees [29].

Several domain-specific MPC protocols have been proposed for federated statistical analysis in economic applications [30,31], public health applications [32-35], and medical applications [36-38]. In particular, Kamm *et al*. [37] proposed to protect privacy in GWAS by requiring a data collection system to secretly share sensitive data among all parties using distributed storage. Similarly, Bogdanov *et al*. [36] proposed a secret-sharing based framework for privacy-preserving statistical data analysis. Xie *et al*. [38] developed another MPC protocol for privacy-preserving genetic association meta-analysis, which allows each site to fully control its local data.

For statistical analysis, a practical alternative (to use cryptographic primitives) is to operate on per-site aggregated statistics; such models do not exchange patient-level data and have produced as accurate results as if data were centralized. With this weaker level of security protection, various statistics and learning computations can be enabled on federated data [39,18,19]. Our approach built on MPC provides stronger security protection regarding per-site statistics.

Data de-identification methods (e.g. suppression or generalization) have been proposed in the database security community to protect the confidentiality of released data on a single site. Such securely released data can be used as input for statistical analysis which automatically preserves privacy. However, utility will be sacrificed due to the noise injected in the released data. In particular, various privacy definitions have been proposed, ranging from *k*-anonymity [40], *l*-diversity [14], to the most recent differential privacy [15]. On the other hand, current privacy policies for data de-identification are inadequate to provide enough protection to genomic data privacy, as reported by the Presidential Commission for the Study of Bioethical Issues [41]. Sweeney *et al*. demonstrated the vulnerability in a recent study that successfully identified the participants of the Personal Genome Project (PGP) [42]. Vaidya *et al*. re-identified a Native American woman from the public query system of the Healthcare Cost and Utilization Project (HCUP) [43]. Like other technologies, the attack models are improving in accuracy and the risk of harm from data disclosure is increasing. Many recent research results show that with some background information about an individual, an adversary can identify or learn sensitive information about the victims from their genomic data [44-47]. NIH has removed most aggregate research results from the public domain due to the potential privacy risks elaborated in [46]. Gymrek *etal*. [45] showed that genomic information can be used to infer an individual's surname. A recent study [48] even demonstrated the possibility of directly inferring a face from an individual's genomic data.

## Methods

### Preliminaries

#### GWAS computation

GWAS are frequently used to map genotypes (the genes within an organism) onto phenotypes (the traits of an organism) [49]. The predominant application for GWAS today is in the study of genetic diseases. GWAS are conducted by examining genetic mutations which differ significantly between individuals who have an illness and those who do not. These individuals are partitioned into the case and control groups, respectively. What follows are brief descriptions of some of the relevant terms in genetics, as well as the statistics we would like to compute over the input data.

*SNPs *In genetics, a DNA sequence consists of multiple nucleotides, where a single nucleotide can take one of four values 'A', 'G', 'T' and 'C'. A Single Nucleotide Polymorphism (SNP) is a DNA sequence variation in which a single nucleotide varies between individuals in a population. Given that nearly 99% human DNA are identical, the study of identifying genetic mutations such as SNPs is essential in determining which genotypes correspond to which human traits. During the past decade, the associations between a number of common diseases (e.g., heart disease, diabetes etc.) and common SNPs have been widely studied [49].

##### Minor allele frequency (MAF)

An allele is a variant of the same gene or the same genetic locus. A minor allele frequency (MAF) is the frequency at which the least frequent allele occurs [49] within a given population. For example, the genotypes of five individuals at the same loci are as follows, AA, AG, AA, AG, and GG. Since G is less frequent than A, and its frequency is $\frac{4}{10}$, The MAF can be calculated as 0.4 in this case.

##### *χ*^2 ^statistic

The *χ*^2 ^statistic is the statistic used by a *χ*^2 ^hypothesis test. Given a set of categories and the frequencies with which observed and expected values fall into those categories, a *χ*^2 ^test can be used to test whether the observed and expected populations differ in a statistically significant way. The *χ*^2 ^statistic is computed as

(1) $$\chi^{2}=\underset{i,j}{\sum }\frac{{\left(obs_{i,j}-exp_{i,j}\right)}^{2}}{exp_{i,j}}$$

where *obs_i,j _*and *exp_i,j _*denote the observed and expected allele counts from allele type *j *(e.g., *j *∈{A,G} in above example) in group *i*(*i*∈ {case and control groups}).

#### PCF based MPC framework

A garbled circuit-based MPC programming framework usually consists of two components: a compiler that compiles a high-level human-readable program into a low-level circuit representation, and a runtime system that executes the circuit distributedly across multiple participant parties. The MPC programming language is designed to be similar to C, though its security guarantees make it more restrictive than C. These include limitations on data input size, lack of support for negative numbers, and inflexible control flow. One particularly salient example is that every loop must run a fixed number of iterations, determined at compile time. This presents challenges in implementing GWAS computations and we will present our solutions to work within those restrictions without sacrificing security protection. In our work, we choose the PCF programming framework [1], which supports garbled circuit-based MPC for two participants. A garbled circuit [50] is a specially designed circuit, which enables two (or more) parties to securely compute a function *f *(*x_A_, x_B_*) without exposing their private secrets (e.g., *x_A _*and *x_B _*are inputs from party A and party B, respectively). The framework includes a compiler called LCCYao, and an execution runtime called BetterYao. Given a C program compiled into bytecode by the LCCYao compiler, the BetterYao runtime executes the circuit representation like any other garbled circuit based protocol [50]: If two parties, say Alice and Bob, want to collaborate in computation, Alice takes the role of the circuit builder. She constructs a circuit (Boolean or arithmetic) to implement the computation algorithm, then generates keys for and encrypts each wire by producing a garbled truth table. Next she sends both the garbled circuit and its inputs to Bob. Bob uses a 1-out-of-2 oblivious transfer[50] to receive this information from Alice. Finally Bob inserts his input values, and runs the circuit on both sets of values. He returns the output to Alice, who can decrypt it since she was the one who generated the keys.

### Design overview

In our system model, we consider two participant institutions which want to conduct GWAS computations in a secure fashion. Each institution holds genomic data of its clients or patients which are deemed sensitive and private. Unlike other personal information which can be changed once disclosed (e.g. credit card numbers), personal genomic data is immutable, which makes it more sensitive and the protection mission-critical. The institutions must comply with various privacy laws and registrations HIPAA [10] which restrict the exchange and sharing of personal identifiable data across institutional boundaries. It is clear that once the personal genome data are disclosed, undesirable consequences may arise. If an insurance company finds out that one of its clients has a mutation making him highly susceptible to a certain disease, then the company might increase the client's premium or even drop the client.

Our proposed privacy-preserving GWAS computations on federated datasets have the following three design goals:

• **Correctness**: The distributed multi-party computation must be able to produce the same result (with allowable round-off error) as a centralized computation performed on a merged version of the federated datasets.

• **Privacy**: The distributed computation must be able to protect the private data of any institution from every other institution.

• **Performance**: The computation must be fast and efficient, making it practical for scalable genomic datasets.

**Figure 1:**
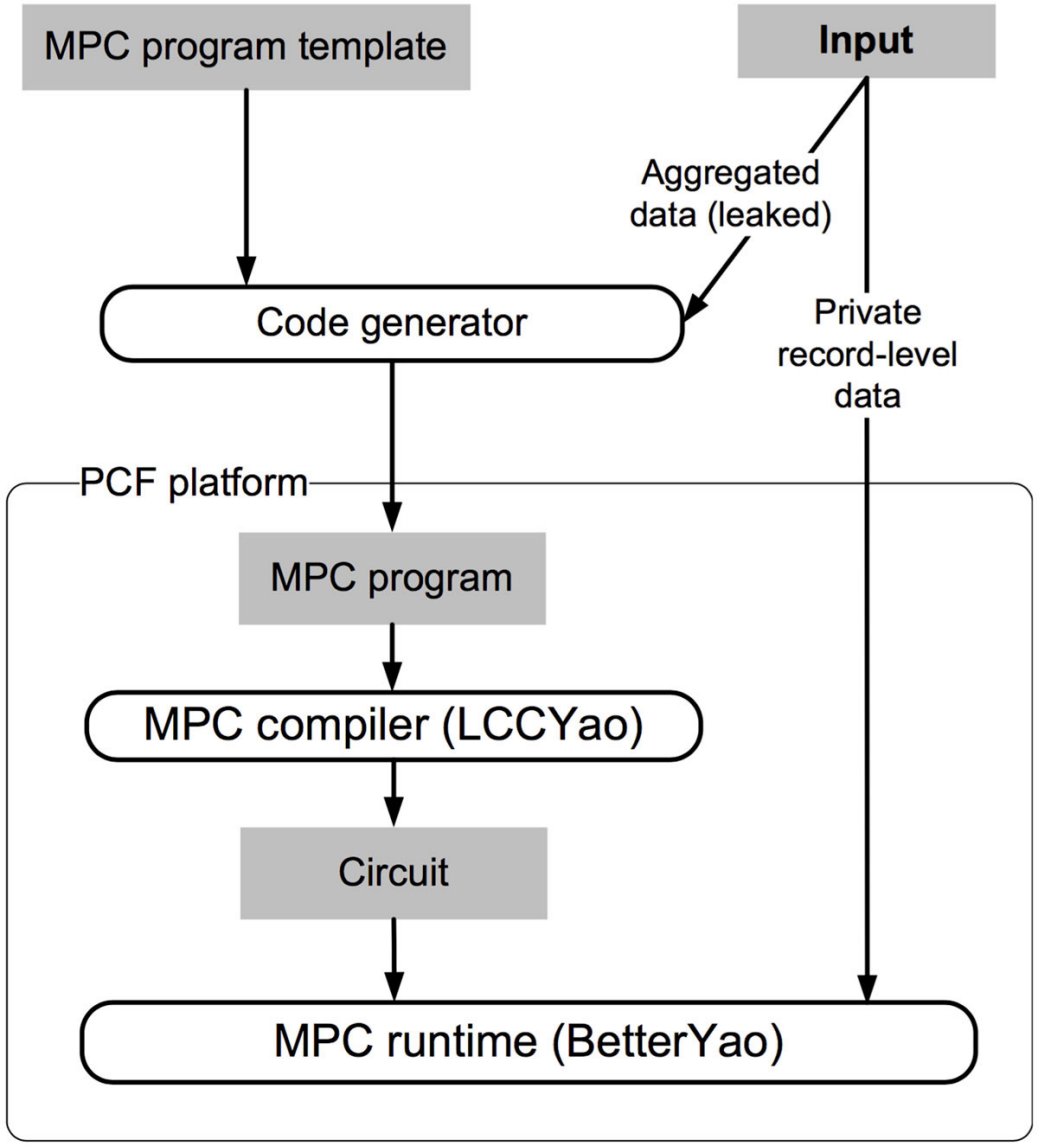
**The system workflow**.

In this work, we focus on the computation of MAF and *χ*^2 ^statistic using the PCF framework. The challenges arise from 1) working within the restrictions inherent in PCF programming model and 2) automating the workflow for optimized performance and ease of management. For the second challenge, the usual workflow of deploying an MPC program (or any computer program in general) is to deploy the executable binary code, configure the runtime system, and then execute the computation. In the BetterYao framework, the maximum input size is very small size (i.e. 8, 000 bits). This requires us to partition the input data properly. The extended workflow is shown in Figure 1, the details of which will be elaborated in the next section.

### Automated work flow

We have implemented the proposed design using PCF from end to end, including input data analysis (using Python), automatic code generation (using Bourne Again SHell or Bash), compilation (using the PCF/LCCYao compiler) and execution (with PCF simulator and BetterYao runtime system). We glue different software components using Bash. The general flow of our secure MPC is enumerated below, and is also illustrated in Figure 1. Note that steps 1-3 and 5 are performed locally within each institution, and step 4 is the mutual MPC performed synchronously by both institutions.

1 **Preprocess the data **Institutions 1 and 2 each have two text files containing the SNPs of *n *individuals over *m *genotypes. One text file corresponds to the case group, the other to the control group. An genotype entry in the data might look like

rs11686243

AG AG AA AG GG AA AG AA GG AG AA AA AA AA ...

where each pair of nucleotides (i.e. "AG") is an SNP of that genotype for one individual.

It is worth noting that not every part of the computation needs to be done by the PCF/BetterYao framework. Only the computational steps which require data from *both *participants needs to be protected by secure MPC. The very first step of the computation, counting the total number of nucleotides of each value ('A', 'G', 'T' or 'C'), can be done locally by each participant. We use a Python script to scan the data files and count the nucleotides for each genotype. The preprocessing tool also captures some important metadata, including the number of individuals whose SNPs are represented, and the total number of genotypes. This metadata is required in step 2. From this point onward, we must rely on secure MPC, since the remainder of the computation simultaneously requires secret data from both parties.

The PCF/BetterYao framework requires that all input data be encoded as hexadecimal text input, no greater than 8000 bits in length. For the sake of efficiency, we assume that typical GWAS will not involve more than 2^15 ^= 32768 participants, so that each nucleotide count input will span more than 16 bits (though this assumption could easily be lifted if necessary). Since each participant has a case and control group for each genotype, each genotype will consume 32 bits of input (16 for the case group, and 16 for the control). Hence each hexadecimal file can contain nucleotide counts for up to $\left\lfloor \frac{8000}{32}\right\rfloor =250$ genotypes.

2 **Generate subset-C code **The PCF/BetterYao system has an interesting restriction. Suppose an eavesdropper listens in on the MPC communication between Institutions 1 and 2. From the amount of time it takes for a mutual computation to complete, the eavesdropper may be able to garner some information about the data being operated on. For this reason, the PCF/BetterYao framework requires that all loops run at a fixed number of iterations, determined at compile time. For purposes of efficiency, we break this assumption by dynamically generating the subset-C code based on the metadata produced in step 1. For instance, we have a line in the code generator

#define TOTAL $total

where $total corresponds to the total number of nucleotides per genotype. The value for $total is provided by the data preprocessor, and is filled in by the code generator. Though this technique may appear to break the security requirement, it will only reveal to the eavesdropper the amount of data that is shared between the participating institutions. We consider this to be a reasonable trade-off for much better performance. This point is further discussed in the "Security" section.

3 **Compile the PCF file **The PCF tool uses the Little C Compiler (LCC) to compile the subset-C code to targeted bytecode, which is then mapped onto a garbled circuit.

4 **Evaluate the circuit **This step is the only one which is performed mutually between institutions. One institution, say Institution 1, is deemed the circuit generator. In our case, the generator will build a Boolean circuit to compute either the MAF or *χ*^2^. Once the circuit has been constructed, all of its wires are then "garbled" according to the Yao protocol[50]. Institution 1 additionally garbles its own input data. Then both the garbled circuit and input are sent to Institution 2, the circuit evaluator. Using a 1-out-of-2 oblivious transfer (OT) protocol[50], Institution 2 similarly garbles its own inputs. With both sets of garbled inputs and the garbled circuit, Institution 2 then proceeds to evaluate the circuit. If the circuit output itself is not garbled, then Institution 2 may report it. Otherwise Institution 1 must report the output.

5 **Report the results **The evaluator is the only party with the cryptographic means to decode the circuit output. In our example, this is Institution1.PCF/BetterYao reports the computation results as hexadecimal output. We provide a script to decode the output into human-readable decimal point form.

### Algorithmic implementation

This section details our algorithms on PCF/BetterYao to compute the MAF and *χ*^2 ^statistics from genotype data.

#### Minor allele frequency

Our algorithm for computing the MAF proceeds by first reading in the count of each nucleotide per case/control group (the output of Step 1 in the previous section), and then determining which is the least frequent and computing its relative frequency.

**Figure 2:**
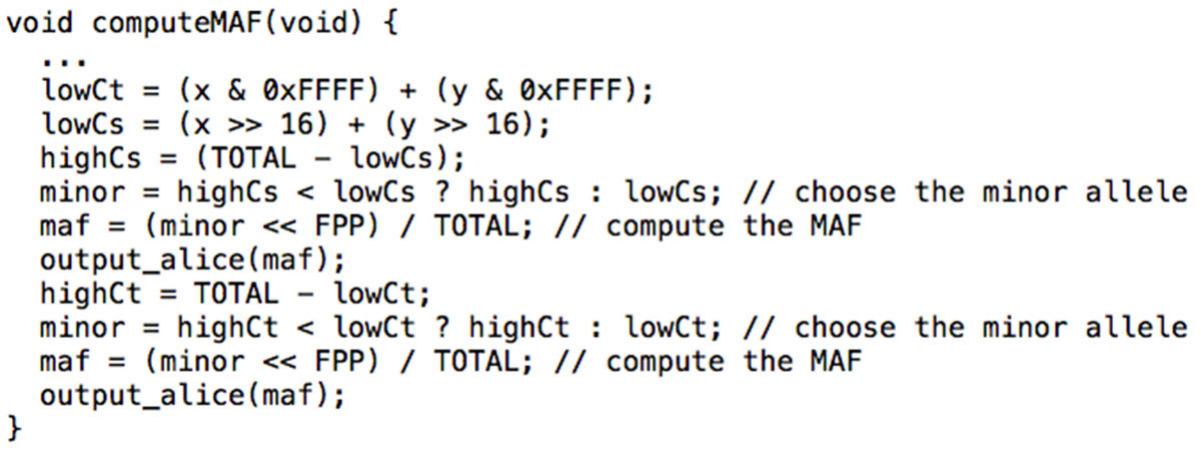
**PCF function to compute minor allele frequency**. Here × and y are the inputs for one genotype read in from Alice and Bob's data, respectively. The low-order 16 bits of each input correspond to a control group, whereas the high-order 16 bits correspond to a case group. We begin by computing the aggregated count of the lexicographically lower (e.g. 'A' < 'G') nucleotide for each group across both parties. lowCt denotes this count for the aggregated control group, and likewise highCt for the control group. The lexicographically high counts are simply the total counts minus the low ones. We then decide which count is lower, and thus must represent the minor allele. Finally we perform a floating point adjustment, divide to obtain the frequency, and output the case and control MAFs to the terminal.

MAFs are computed and reported separately for the case and control groups. Once all of the nucleotides have been counted, we determine which allele is minor (i.e. with smaller frequency). We then compute the frequency of the minor allele, and emit it to Alice's terminal. Note that the PCF tool has no native support for primitive types other than unsigned 32-bit integers, which forces us to simulate floating point computation in order to divide. This is accomplished by shifting the dividend to the left by *FPP *bits before dividing, where *FPP *is the desired Floating Point Precision. For our purposes, we let *FPP *= 16. A sample of the algorithm implementation is given in Figure 2.

#### *χ*^2 ^statistic

**Figure 3:**
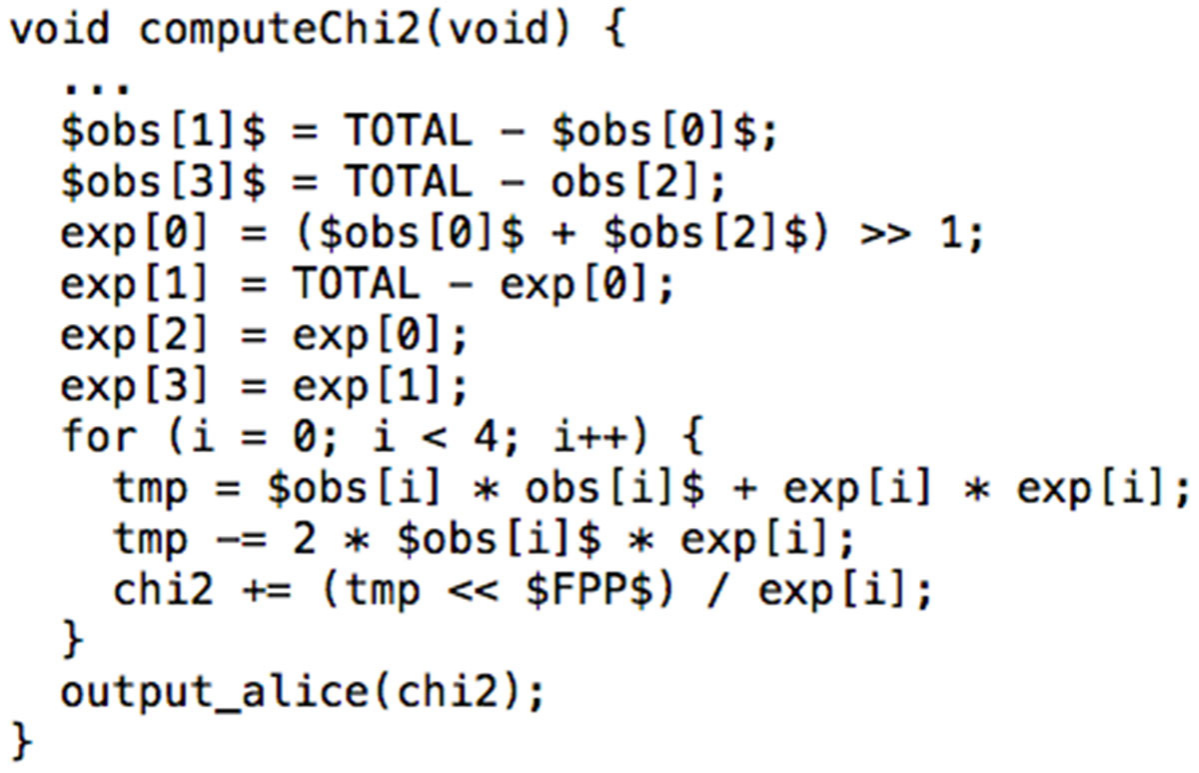
**Computing *χ*^2 ^statistic**. *obs*[0] and *obs*[1] are the case group's low and high allele counts, respectively, with *obs*[2] and *obs*[3] as the respective counts for the control group. *exp*[0 ... 3] are the respective expected counts for each allele in each group. The "for" loop computes the *χ*^2 ^statistic according to Equation 1.

The process for computing the *χ*^2 ^statistic proceeds by first reading in the number of alleles per group, and then computing *χ*^2 ^as in Equation 1. The computation of *χ*^2 ^is illustrated in Figure 3. One implementation note is that since PCF does not support signed integer computation, we must be very careful to avoid any expression where a negative number may result. Indeed, this could happen in the numerator of Equation 1 if *obs *<*exp*. To avoid a signed overflow, we observe that (*obs−exp*)^2 ^= *obs*^2 ^+*exp*^2^*−*2·*obs*·*exp *and use this substitution in our computation. This guarantees that at no point will we compute a negative number.

### Parallelism

Perhaps the most substantial optimization we were able to make was to parallelize our MAF and *χ*^2 ^statistic computations. PCF and BetterYao have no native support for parallelization. However, since computations across genotypes are independent, we were able to break up the input into many smaller files, and spawn a child process for each file.

In fact, partitioning the input is mandated by BetterYao's restriction on input data containing greater than 8, 000 bits of information. For example, our sample dataset contains 311 genotypes for 400 individuals. We were able to compute the nucleotide counts for each case/control group and genotype in a preprocessing step. We were then able to compress the counts for up to 250 genotypes into 8, 000 bits. Our largest dataset spanned 9, 330 genotypes. Hence we had to partition the entire dataset into $\left\lceil \frac{9,330}{250}\right\rceil =38$ input files. Our data preprocessor performs the correct partitioning automatically.

Once *n *input files have been produced, *n *child processes are created, one process per input file. When the dataset is large enough, there may be hundreds of input files, and thus hundreds of processes. On commodity hardware this can be disastrous for the overall running time, due to high memory overhead and poor cache performance. To address this issue, we enqueue all of the processes, allowing only a few to run at any given time. We found that for a system with *c *CPU cores, executing 2*c *processes simultaneously would best optimize the overall running time.

## Results: Performance and security analysis

### Experiment setup

Our test setup includes two separate machines located on the same network at Syracuse University. Both machines have 6GB of RAM and Intel Core i5 750 Processors with 4 physical cores, each clocked at 2.66 GHz. Each machine is running Ubuntu 12.04 LTS. The experimental data consist [51] of the SNPs for 311 genotypes taken from 200 individuals in a case group and another 200 individuals in a control group. The case and control individuals were extracted from Personal Genome Project (PGP) participants and HapMap CEU individuals, respectively. In our experiment, both case and control datasets were evenly partitioned between two institutions. Table 1 illustrates the distribution of case and control individuals.

**Table 1:** Distribution across institutions for the sample data.

	Institution 1	Institution 2
Case	100	100

Control	100	100

### Performance results

**Figure 4:**
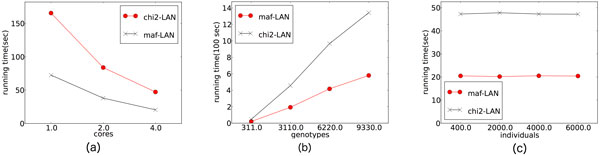
**Execution time for evaluating MAF and *χ*^2 ^statistics with different experimental setups**.

Figure 4 shows the performance results in terms of execution time in minutes under different experimental setups. Each subfigure illustrates the impact of different experimental parameters on the system performance. In Figure 4(a), we run the MAF and *χ*^2 ^statistic calculation on the sample dataset by varying the number of usable CPU cores. By increasing the number of cores from 1 to 4, we observed roughly 3.4 times speedup for both MAF and *χ*^2^statistic computations. With all four cores, we were able to achieve an execution time of 20.49 seconds for MAF, and 47.27 seconds for *χ*^2^.

Figure 4(b) shows how our implementation scales with respect to the number of genotypes in the dataset. Since we only had access to the sample dataset with311 genotypes, we replicated this data 10, 20, and 30 times to simulate much larger datasets. With respect to the number of genotypes the observed running times scaled linearly. For MAF, increasing the number of genotypes from 311 to 3110 (a 10x increase) increased the running time from roughly 20 seconds to 193 seconds (a 9.4x increase). On the largest dataset with 9330 genotypes, we achieved a running time of 9 minutes, 40 seconds for MAF, and 22 minutes, 22 seconds for *χ*^2^. The *χ*^2 ^computation consistently consumes more execution time because it requires more multiplication and division, which are expensive operations in Boolean circuits.

When varying the number of individuals in the case and control groups as in Figure 4(c), the results are invariant. This is because in both MAF and *χ*^2^, the alleles are counted during the offline stage which does not use garbled circuits, and hence performs substantially better. In fact, increasing the number of individuals by a factor of 15 only increased the offline computation time by 0.24 seconds. The online computation time was unaffected. As before, we replicated individual records in the sample dataset to simulate several large datasets.

**Figure 5:**
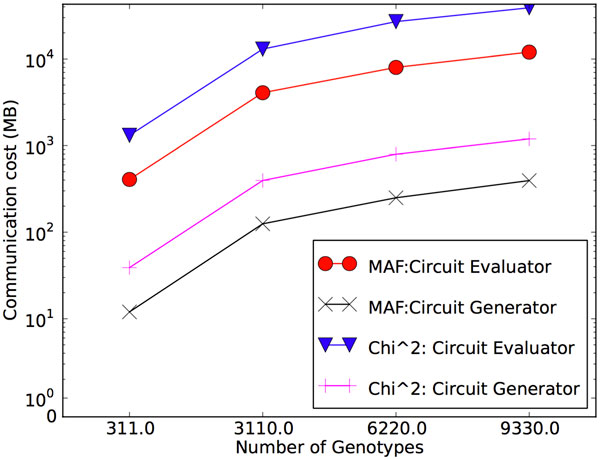
**Total bytes transferred by each party, with respect to the number of individuals in the merged datasets**.

Figure 5 provides the communication costs associated with each computation, relative to the number of genotypes in the input data set. For each of MAF and *χ*^2^, we provide the total number of bytes transferred by each party. Most notably, the circuit evaluator yields at least an order of magnitude more data than the circuit generator. Varying the number of individuals had no measurable impact on the network toll, again since this factor only impacts the offline computation. Data transfers while computing the *χ*^2 ^statistic were consistently 3-4 times greater than they were during a MAF computation. This is because the garbled boolean circuits that implement multiplication and division (as required by *χ*^2^) are more complex than those that implement addition and subtraction (more often used when computing MAF).

### Security analysis

We analyze the security property, specifically privacy preservation, of our federated GWAS computation framework. The security guarantees made by our system are almost entirely dependent upon the underlying MPC runtime protocol. The PCF/BetterYao protocol can support a variety of security models, ranging from the semi-honest model [23] (which assumes all participant parties will not do anything more than peeking into the data flowing through themselves; they will behave according to what is required by the protocol) to the more hostile malicious model (which assumes participant parties can act at their own will without restriction from the protocol).

Note that our design, as illustrated in Figure 1, leaks aggregated information about per-party input data. Specifically, each participating party discloses the number of genotypes and the number of individuals in the input. We argue that these two pieces of information only reveal very coarse-grained information about the input data; the input data size can be inferred from the computation time anyway. The design of leaking the information is mainly for the purpose of performance optimization. We always present an alternative to the institutional users, that is, an institution which is not willing to disclose that information can simply use the least upper bound of all of its data as a fixed size for all computations. This way all computations will run in the same amount of CPU time.

## Discussion on limitations

In this paper, we experimented with the PCF circuit framework [1] and BetterYao runtime system. The proposed framework is able to generate customized circuits using C-like programs for secure two-party computation on federated genomic datasets. In our current implementation, the proposed framework supports both secure and accurate MAF counting and *χ*^2 ^statistic computation between two parties. We have conducted a comprehensive performance evaluation in terms of scalability of data size, parallel computation over multiple cores, and different network topologies. The experimental results show that the proposed framework provides promising performance in both computational tasks. For example, it only takes a few minutes to securely compute the *χ*^2 ^statistics over thousands of genotypes between case and control groups.

It is worth mentioning that the proposed framework still has some limitations. First, as the current implementation only supports secure two-party computation, only two hospitals or institutions are allowed to collectively conduct federated analysis with our implementation. This limitation is inherited from that of the BetterYao framework. We are actively planning to extend our work to allow more than two-party data analysis. This goal can be achieved by using other secure multi-party platforms (e.g. FairplayMP [23]) with the additional costs of computation and communication and by re-engineering our implementation for more general scenarios.

Another limitation is the noticeable computational and communication overhead in the current implementation, when comparing the cost to carry out the same computation in a centralized non-encrypted environment. Owing to the extensive use of cryptographic primitives in MPC and communication overhead through the Internet, over which federated analysis is deployed, the performance may become a bottleneck when handling extra large scale genomic data. However, the proposed framework demonstrated good scalability for parallel computing in a multi-core system. This warrants further investigation along this line to improve the privacy-preserving federated genomic data analysis using secure MPC.

## Competing interests

The authors declare that they have no competing interests.

## Authors' contributions

YT conceived of the study and oversaw all development. SDC implemented and optimized the MAF and *χ*^2^algorithms in PCF, and drafted the manuscript. XJ and SW contributed to the background information on GWAS. SC serves as SDC's academic advisor at Syracuse University. All authors contributed to the final manuscript.

## Declarations

Publication funding for this supplement was supported by iDASH U54HL108460, iDASH linked R01HG007078 (Indiana University), NHGRI K99HG008175 and NLM R00LM011392.
